# RNA-Seq Analyses Reveal That Endothelial Activation and Fibrosis Are Induced Early and Progressively by *Besnoitia besnoiti* Host Cell Invasion and Proliferation

**DOI:** 10.3389/fcimb.2020.00218

**Published:** 2020-05-15

**Authors:** Alejandro Jiménez-Meléndez, Chandra Ramakrishnan, Adrian B. Hehl, Giancarlo Russo, Gema Álvarez-García

**Affiliations:** ^1^SALUVET, Animal Health Department, Faculty of Veterinary Sciences, Complutense University of Madrid, Madrid, Spain; ^2^Institute of Parasitology, University of Zurich, Zurich, Switzerland; ^3^Functional Genomics Center Zurich, Zurich, Switzerland

**Keywords:** *Besnoitia besnoiti*, tachyzoite, primary bovine aorta endothelial cells (BAEC), transcriptome, RNA-Seq

## Abstract

The pathogenesis of bovine besnoitiosis and the molecular bases that govern disease progression remain to be elucidated. Thus, we have employed an *in vitro* model of infection based on primary bovine aortic endothelial cells (BAEC), target cells during the acute infection. Host-parasite interactions were investigated by RNA-Seq at two post-infection (pi) time points: 12 hpi, when tachyzoites have already invaded host cells, and 32 hpi, when tachyzoites have replicated for at least two generations. Additionally, the gene expression profile of *B. besnoiti* tachyzoites was studied at both pi time points. Up to 446 differentially expressed *B. taurus* genes (DEGs) were found in BAEC between both pi time points: 249 DEGs were up-regulated and 197 DEGs were down-regulated at 32 hpi. Upregulation of different genes encoding cytokines, chemokines, leukocyte adhesion molecules predominantly at 12 hpi implies an activation of endothelial cells, whilst upregulation of genes involved in angiogenesis and extracellular matrix organization was detected at both time points. NF-κB and TNF-α signaling pathways appeared to be mainly modulated upon infection, coordinating the expression of several effector proteins with proinflammatory and pro-fibrotic phenotypes. These mediators are thought to be responsible for macrophage recruitment setting the basis for chronic inflammation and fibrosis characteristic of chronic besnoitiosis. Angiogenesis regulation also predominated, and this multistep process was evidenced by the upregulation of markers involved in both early (e.g., growth factors and matrix metalloproteinases) and late steps (e.g., integrins and vasohibin). *Besnoitia besnoiti* ortholog genes present in other Toxoplasmatinae members and involved in the lytic cycle have shown to be differentially expressed among the two time points studied, with a higher expression at 32 hpi (e.g., ROP40, ROP5B, MIC1, MIC10). This study gives molecular clues on *B. besnoiti*- BAECs interaction and shows the progression of type II endothelial cell activation upon parasite invasion and proliferation.

## Introduction

*Besnoitia besnoiti* is the ethiological agent of bovine besnoitiosis (Besnoit and Robin, 1912), a re-emerging disease in Europe with a progressive dissemination in beef cattle herds and negative impact in cattle welfare and fertility (European Food Safety Authority, 2010; Cortes et al., 2014). This parasitic disease is responsible for both cutaneous and systemic clinical signs, as well as sterility in bulls (Álvarez-García et al., 2014). Disease initiates with the acute stage, when the tachyzoites are fast-replicating in endothelial cells, and evolves with the chronic stage, characterized by the development of bradyzoite-containing tissue cysts located mainly in the subcutaneous tissue and mucous membranes. Acutely infected animals can develop oedemas, orchitis, respiratory distress, and may suddenly die due to a cardio-respiratory failure. Chronically infected cattle develop pathognomonic tissue cysts responsible for skin lesions and the initial orchitis may end up with testis atrophy (reviewed by Álvarez-García et al., 2014).

Endothelial cells (ECs) are one of the main target cells for parasite replication during the acute infection (McCully et al., 1966), and their dysfunction may be determinant for tissue damage, particularly in testes and lungs. Vascular damage has been histologically described mainly in skin, characterized by degenerative and fibrinoid necrotic vascular lesions, vasculitis and thrombosis in medium-sized and small veins, as well as small arteries (Pols, 1960; McCully et al., 1966; Basson et al., 1970; Bigalke et al., 2004). The molecular bases that underlies these host-parasite interactions remain to be elucidated. *In vitro* studies performed so far in calf umbilical vein endothelial cells (BUVEC) (Maksimov et al., 2016; Taubert et al., 2016) showed that *B. besnoiti* infection results in an increase in the transcripts of key genes such as P-selectin, intercellular adhesion molecule 1 (ICAM-1), chemokines (CXCL1, CXCL8, CCL5), IL-6 and COX-2 related to endothelial cell activation and leukocyte recruitment.

As obligate intracellular pathogens, protozoan parasites belonging to Toxoplasmatinae subfamily (subphylum Apicomplexa) have developed unique adaptations that allow them to modify the host cellular machinery to alter host gene expression and favor their replication and dissemination known as lytic cycle (Blader et al., 2015; Hakimi and Bougdour, 2015). However, time points of adhesion/invasion, proliferation and egress of *B. besnoiti* tachyzoites differ from the ones reported in other Toxoplasmatinae parasites, since tachyzoites showed a delayed invasion of host cells, followed by a lag phase of up to 24 hpi, as well as the capacity to survive for up to 24 hpi when still extracellular (Frey et al., 2016). Moreover, species-specific differences in the protein repertoire involved have been reported between *Toxoplasma gondii* and *Neospora caninum* (Reid et al., 2012). Proteins contained in specific apicomplexan organelles such as rhoptries (ROP), dense granules (GRA) or micronemes (MIC) (Blader et al., 2015) play a key role at a transcriptional level altering many phenomena (e.g., blockage of host immune pathways, activation of transcription factors, modifications on the chromatin and small non-coding RNA) (Hakimi et al., 2017). In *B. besnoiti*, the identification of ortholog genes has been hampered by the lack of the whole genome sequence (García-Lunar et al., 2013, 2014), which has only been recently announced (Schares et al., 2017).

Herein, we aimed to dissect the molecular mechanisms that govern the endothelial injury during acute *B. besnoiti* infection in a recently standardized *in vitro* model based on primary target bovine aorta endothelial cells (BAEC) by means of RNA-Seq. Remarkably, this cell line was free from bovine viral diarrhea virus (BVDV), which is widely known to alter the transcriptomic profile of endothelial cells *in vitro* (Neill et al., 2008) and to facilitate early *B. besnoiti* invasion (Jiménez-Meléndez et al., 2019). Transcriptomics analyses were carried out at two post-infection (pi) time points, representative of early invasion (12 hpi) and intracellular proliferation (32 hpi). In parallel, a repertoire of *B. besnoiti* tachyzoite differentially expressed genes (DEG) involved in the lytic cycle were also identified.

## Materials and Methods

### Parasites, Cell Cultures, and Experimental Design

The primary bovine aortic endothelial cells (BAEC) were maintained in M200 medium (Gibco, Life Technologies, Thermo Fisher Scientific, Waltham, MA, USA) containing 20% of FBS, 100 IU/mL of penicillin, 100 mg/mL of streptomycin, 0.25 μg/mL amphotericin B and Large Vessel Endothelial Growth supplement as described by Jiménez-Meléndez et al. (2019). Tachyzoites from the Bb-Spain 1 isolate of *B. besnoiti* were routinely maintained in a monolayer culture of the monkey kidney MARC-145 cell line as previously described (Jiménez-Meléndez et al., 2017). As quality controls, the absence of *Mycoplasma* spp. and BVDV were checked according to Jiménez-Meléndez et al. (2019).

Tachyzoites were harvested at 3 days post infection (dpi), when the majority of parasites were still intracellular, by recovering the infected cell monolayer with a cell scraper, followed by repeated passages through a 25-gauge needle at 4°C and separation from cell debris on a PD-10 column (Frey et al., 2016). Tachyzoite viability was confirmed by trypan blue exclusion followed by counting in a Neubauer chamber. Purified tachyzoites were used to infect BAEC confluent cell monolayers as described below.

Infections in BAEC for transcriptomics analyses were performed as previously described (Jiménez-Meléndez et al., 2019). T25 flasks seeded with confluent BAEC monolayers (2 × 10^6^ cells) were infected with purified tachyzoites at a multiplicity of infection (MOI) of 10. Preliminary experiments were performed in order to optimize the infection rate by increasing MOIs (5, 10, 20). The maximum invasion rate for the isolate employed was 20% (unpublished data). Infected flasks were washed at 12 hpi, when most parasites have already invaded but have not yet started replicating, and at 32 hpi, when parasites have replicated at least twice (Jiménez-Meléndez et al., 2019). Cells and parasites were recovered using a cell scraper in 5 ml of cold PBS, pelleted by centrifugation at 1,350 × g for 10 min, resuspended in 300 μl of RNAlater® (Thermo Fisher Scientific, Madrid, Spain) and stored at −80°C until RNA extraction. In parallel, T25 flasks containing non-infected BAECs were similarly collected at 12 hpi. All analyses were performed with four biological replicates and, for each replicate, technical replicates were collected for further validation by quantitative real time PCR (qPCR).

### RNA Extraction

Total RNA from the cell culture samples was extracted using QIAGEN RNeasy Mini Kit following a QIAshredder® homogenization according to the manufacturer's instructions. Briefly, samples in RNAlater® were lysed in 900 μL of RLT buffer + β-mercaptoethanol at 10 μl/mL and column purified; an on-column DNase I digestion was included. Total RNA was eluted in RNase-free water. The RNA concentration and purity were assessed spectrophotometrically at 260 nm using NanoDrop 1000 (Implen, Munich, Germany).

### Quality Control of Total RNA, Library Preparation, and RNA-Seq

The quality and quantity of the total RNA were further determined in an Agilent TapeStation 4200 to determine the RNA integrity Number (RIN). Only RNA extracts with concentrations between 0.5 and 5 μg/mL, RINs higher than 9.6, and with 260/280 ratios between 1.8 and 2.0 were included in the study. The TruSeq RNA Sample Prep Kit v2 (Illumina, Inc., California, USA) was used in the succeeding steps. Briefly, mRNA was enriched from total RNA samples (100–1,000 ng) using poly-A selection before transcription into double-stranded cDNA. The cDNA samples were fragmented, end-repaired and polyadenylated before ligation of TruSeq adapters containing the index for multiplexing Fragments containing TruSeq adapters on both ends were selectively enriched with PCR. The quality and quantity of the enriched libraries were validated using the Qubit® (1.0) Fluorometer and the Caliper GX LabChip® GX (Caliper Life Sciences, Inc., USA). The product is a smear with an average fragment size of ~260 bp. The libraries were normalized to 10 nM in 10 mM Tris-Cl/0.1% Tween 20, pH8.5.

The TruSeq PE Cluster Kit v3-cBot-HS or TruSeq SR Cluster Kit v3-cBot-HS (Illumina Inc.) was used for cluster generation using 10 pM of pooled normalized libraries on the cBOT. Stranded sequencing was performed on the Illumina HiSeq 4000 with single end sequencing at 125 bp using the TruSeq SBS Kit HS4000 (Illumina Inc.).

### Computational Analysis of RNA-Seq Data

Reads were quality-controlled with FastQC. Sequencing adapters were removed with Trimmomatic (Bolger et al., 2014), and reads were hard-trimmed by 5 bases at the 3' end. Successively, reads at least 20 bases long, and with an overall average phred quality score >10 were aligned to the reference genome and transcriptome of *Bos Taurus* (UCSC/bosTau7) and *B. besnoiti* (BbLIS14 in-house assembled genome, unpublished) with STAR v2.5.1 (Dobin et al., 2013), with default settings for paired end reads.

Distribution of the reads across genomic isoform expression was quantified using the R package GenomicRanges (Lawrence et al., 2013) from Bioconductor Version 3.0. Differentially expressed genes were identified using the R package DESeq2 (Love et al., 2014) from Bioconductor Version 3.0. DESeq2 performs an internal normalization, where the geometric mean is calculated for each gene across all samples. The counts for a gene in each sample is then divided by this mean. The median of these ratios in a sample is the size factor for that sample. We used a threshold of ≥1.5fold difference (fold change) in mRNA levels (measured as normalized averaged mapped sequence reads per gene) and a false discovery rate (FDR) ≤ 0.05 in order to categorize genes as differentially expressed (DE).

### Functional Enrichment and Network Analysis

Functional enrichment was applied to all comparisons performed using the genome from *Bos taurus* available at the Panther website (http://www.pantherdb.org/) and the statistical overrepresentation test, using an FDR of 0.05 as threshold. The outcome of the gene ontology (GO) analysis was used for semantic clustering using REVIGO in order to identify non-redundant GO terms (Supek et al., 2011). The analyses were performed with an allowed similarity of 0.7 both for up-regulated and down-regulated genes at both infection time points, and with default settings in advanced options.

Furthermore, STRING 10 version 11.0 (Szklarczyk et al., 2015) was used to identify interesting associations between the significant genes identified in our study and to construct Kyoto Encyclopedia of Genes and Genomes (KEGG) pathways (Kanehisa, 2019). Using the STRING database (http://string-db.org/), multiple proteins were chosen from the website interface setting the maximum relevance to 0.9. The differentially expressed genes (DEGs) names were inserted as the input in the list of names, and *Bos taurus* was chosen as the organism.

Further annotation and GO assignment in *Besnoitia besnoiti* were performed using the Blast2GO platform and NCBI's nr protein database. Blast2GO assigns descriptions using a language processing algorithm to hit descriptions, which extracts informative names and avoids low-content terms (Conesa and Gotz, 2008). These descriptions were manually curated where necessary.

### Quantitative Real-Time PCR (qPCR) for Transcriptome Validation

Reverse transcription was performed using the master mix SuperScript® VILO™ cDNA Synthesis Kit (Invitrogen, Paisley, UK) in a 20 μL reaction using up to 2.5 μg of total RNA. cDNA was sequentially diluted to 1:20, 1:80, 1:320 and 1:1,280, and all dilutions were analyzed by quantitative real-time PCR (qPCR). Quantitative real-time PCRs were performed in 25 μL volumes using 12.5 μL of Power SYBR®PCR Master Mix (Applied Biosystems, Foster City, CA, USA), 10 pmol of each primer and 5 μL of the diluted cDNA samples. Reactions were performed in an ABI 7500 FAST Real Time PCR System (Applied Biosystems, Foster City, CA, USA). Relative expression was calculated using the comparative method 2^−ΔΔCt^ (Livak and Schmittgen, 2001) after normalization with housekeeping genes actin β (ACTB) (ENSBTAG00000026199) and separately GAPDH (ENSBTAG00000014731) for bovine genes (Puech et al., 2015; Horcajo et al., 2017) and BbACT1 and GAPDH1 for the *B. besnoiti* genes (BbLIS14 in-house assembled genome, unpublished). The primers used to amplify the target genes are listed in Supplementary Tables 1, 2.

## Results and Discussion

### Sequence Mapping and Quality of RNA-Seq Data

More than 240 million reads were produced in total with 12 different samples. After alignment, an average of 90% of the high-quality reads mapped to the reference *B. taurus* genome, which is a global indicator of sequencing accuracy. Approximately 0.174 % of the total RNA population was attributable to *B. besnoiti*, with a higher percentage of mapped reads to *B. besnoiti* in samples collected at 32 hpi (Supplementary Table 3). To study reproducibility and the experimental variation between replicates, normalized RNA-Seq data were subjected to principal component analysis (PCA), showing that all samples involved in the present work clustered into three biologically distinct groups (Supplementary Figure 1).

Genes expressed commonly associated with ECs, such as thrombospondin, PECAM1 (CD31), Vimentin or Endothelial Specific Molecule (ESM-1), were found, with mean signals above 46.6k normalized counts in all samples confirming the validity and quality of the uninfected control sample.

### Host Cell Transcriptome Modulation by *B. besnoiti* Tachyzoites

Our results showed an early BAEC modulation at 12 hpi characterized by a proinflammatory and procoagulant state upon *B. besnoiti* invasion and a progression of endothelial activation with upregulation of fibrosis markers along the lytic cycle up to 32 hpi. The gene description and fold change values of a selection of *Bos taurus* differentially expressed genes (DEGs) at each time point studied are shown in Table 1.

**Table 1:** Gene description and fold change values of a selection of *Bos taurus* differentially expressed genes (DEGs) at each time point studied.

	**Gene**	**Description**	**FC 12 hpi**	**FC 32 hpi**	**FC 32 hpi**
			vs. C-^*^	vs. C-^*^	vs. 12 hpi^*^
Endothelium protection	NOX5	NADPH oxidase 5. It is involved in ROS production, proliferation, and formation of capillary-like structures, contributing to the endothelial response to thrombin.	−2.74	1.24	3.40
	SOD3	Superoxid dismutase 3. It is a secreted enzyme responsible for the redox balance in specific tissues, including ECs, preventing oxidative damage and preserving nitric oxide (NO) availability.	−2.70	−1.55	1.74
	SERPIN5	Serpine 5. It inactivates serine proteases by binding irreversibly to their serine activation site. It is involved in the regulation of intravascular and extravascular proteolytic activities.	−2.49	1.20	2.97
Extracellular matrix organization	EPAS1	Endothelial PAS domain-containing protein 1. It is an important regulator of vascularization, maybe involved in the regulation of endothelial cell gene expression in response to hypoxia.	−2.50	−2.54	−1.01
	ECM2	Extracellular matrix protein 2. It promotes matrix assembly and cell adhesiveness.	−2.45	−1.20	2.06
	ADAMTS1^a^	It is an active metalloprotease, which may be associated with various inflammatory processes and crossing of biological barriers.	14.55	5.91	-2.44
	MT1A	Metallothionein 1A. Metallothioneins have a high content of cysteine residues that bind various heavy metals.	1.28	6.23	4.83
	MMP14^a^	Matrix Metalloproteinase 14. Endopeptidase that degrades various components of the extracellular matrix, such as collagen. It acts as a positive regulator of cell growth and migration.	1.37	−1.20	−1.65
	ITGA2^a^	Integrin alpha-2/beta-1 is a receptor for laminin, collagen, collagen C-propeptides, fibronectin and E-cadherin. It is responsible for adhesion of platelets and other cells to collagens, modulation of collagen and organization of newly synthesized extracellular matrix.	1.35	−.83	−2.45
	ITGA5^a^	The alpha-V (ITGAV) integrins are receptors for vitronectin, cytotactin, fibronectin, fibrinogen, laminin, matrix metalloproteinase-2, osteopontin, osteomodulin, prothrombin and thrombospondin.	1.35	1.33	−1.01
	ITGA10^a^	Integrin alpha-10/beta-1 is a receptor for collagen.	−1.41	−3.09	−2.18
	ITGB8^a^	Integrin alpha-V:beta-8 is a receptor for fibronectin.	−1.44	1.47	2.12
	CLD1	Claudin-1 is a key component of the tight junction complexes, regulating the permeability of epithelia. Also, it has been shown an important role of claudin-1 in wound healing responses.	−1.06	2.41	2.24
Activation of endothelial cells and leukocyte recruitment	SELE	Selectin E. It is a cell-surface glycoprotein having a role in immune adhesion. It mediates in the adhesion of blood neutrophils in cytokine-activated endothelium through interaction with SELPLG/PSGL1.	2.00	−3.76	−7.50
	SELP	Selectin P. It mediates the interaction of activated endothelial cells or platelets with leukocytes.	2.10	−1.77	−3.70
	VCAM-1	Vascular Cell Adhesion Molecule 1. It is crucial in cell-cell recognition: e.g., leukocyte-endothelial cell adhesion, interacting with integrins.	−1.14	−2.71	−2.37
	ICAM-1	Intercellular Cell Adhesion Molecule 1. ICAM proteins are ligands for leukocyte adhesion proteins mediated through integrins.	1.43	−1.68	−2.38
	IL-1A	Interleukin 1A. IL-1 cytokines are involved in the inflammatory response, being identified as endogenous pyrogens and potent proinflammatory proteins.	1.61	−3.85	−6.18
	IL-6	Interleukin 6. It is a potent inductor of the acute phase response of inflammation.	13.71	1.17	−11.27
	CCL2	C-C motif chemokine 2. It acts as a ligand for C-C chemokine receptor CCR2. It signals through binding and activation of CCR2, exhibiting a chemotactic activity for monocytes and basophils. It is a profibrotic marker.	2.13	−2.00	−4.25
	CCL24	C-C motif chemokine ligand 24. It is also known as eotaxin 2. It exhibits a chemotactic activity for eosinophils, priming them for the release of TNFa, TGFb and IL6, implicated in epithelial damage and microvascular leakage.	2.94	−2.83	−8.16
	CXCL2	C-X-C motif chemokine ligand 2. It is also known as Growth-regulated protein homolog beta (GROβ). It has chemotactic activity for neutrophils acting after CXCL-1, also implicated in the branching of endothelial cells.	2.71	−4.21	−11.32
	CXCL3	C-X-C motic chemokine ligand 3. It is also known as Growth-regulated protein homolog gamma (GROγ). It has chemotactic activity for neutrophils. It may play a role in inflammation and exert its effects on endothelial cells in an autocrine manner.	1.40	−2.20	−3.06
Coagulation	PLAUR	Urokinase plasminogen activator surface receptor. It plays a role in localizing and promoting plasmin formation and mediates the proteolysis-independent signal transduction activation effects of U-PA.	1.99	−1.45	−2.86
	PLAT	Tissue-type plasminogen activator converts plasminogen to plasmin, playing an important role in tissue remodeling and degradation, as well as in cell migration.	1.96	−1.66	−3.24
	FN1^a^	Fibronectin binds cell surfaces with ECM components such as collagen, fibrin, heparin, DNA, and actin. It is involved in cell adhesion, cell motility, wound healing, and maintenance of cell shape.	1.21	4.25	3.50
Fibrosis	THBS1	Thrombospondin-1. It is an adhesive glycoprotein that mediates cell-to-cell and cell-to-matrix interactions, binding to heparin. It presents antiangiogenic properties.	2.53	2.57	1.02
	MCSF-1	Macrophage colony-stimulating factor 1. It is a cytokine that plays an essential role in the regulation of survival, proliferation and differentiation of hematopoietic precursor cells. It promotes the release of proinflammatory chemokines.	1.25	−2.57	−2.04
	VASH-1^a^	Vasohibin 1. Vasohibin-1 (VASH1) was recently discovered as a novel endothelium-derived negative feedback regulator of vascularization.	−1.22	1.35	1.65
	HBEGF^a^	Proheparin-binding EGF-like growth factor. Growth factor that mediates its effects via EGFR, ERBB2 and ERBB4. It is mitogenic for fibroblasts.	1.92	−1.11	−2.11
TNFα/ NF-κβ signaling	TNFAIP1	TNF alpha induced protein 1. It mediates the proinflammatory effects of tumor necrosis factor alpha (TNFα).	−1.02	−1.51	−1.53
	TNFRSF19	Tumor necrosis factor receptor superfamily member 19. It can mediate the activation of JNK and NF-κβ signaling pathways.	−2.25	1.34	3.00
	TNFRSF1B	Tumor necrosis factor receptor superfamily member 1B. It is a receptor with high affinity for TNFα, which mediates most of the metabolic effects of TNFα.	−1.07	1.41	1.50
	NFKB2	It is also known as p100, processed to p52. NF-kappa-B is a pleiotropic transcription factor present as a homo- or heterodimeric complex formed by the Rel-like domain-containing proteins RELA/p65, RELB, NFKB1/p105, NFKB1/p50, REL and NFKB2/p52. The NF- κβ heterodimeric RelB-p52 complex is a transcriptional activator, whilst the NF- κβ p52-p52 homodimer is a transcriptional repressor.	−1.17	−1.31	−1.53
	NFKBIA	NF-kappa-B inhibitor alpha (IKBA). It inhibits the activity of dimeric NF- κβ/REL complexes by trapping REL dimers in the cytoplasm. Upon proinflammatory responses it becomes phosphorylated, promoting its degradation and the translocation of dimeric RELA to the nucleus.	1.40	−1.35	−1.88
	NFKBIE	NF-kappa-B inhibitor epsilon (IKBE). It inhibits NF- κβ by complexing with and trapping it in the cytoplasm. It inhibits DNA-binding of NF- κβ p50-p65 and p50-c-Rel complexes.	1.43	−1.55	−2.20
	NFKBIZ	NF-kappa-B inhibitor zeta (IKBZ). It is involved in NF-kappa-B transcription factor complexes regulation. It inhibits NF- κβ activity without affecting its nuclear translocation upon stimulation, but it has been also shown to present a transcriptional activation activity, being involved in the induction of inflammatory genes activated through TLR/IL-1 receptor signaling.	1.68	−2.24	−3.76

a *Genes also involved in angiogenesis*.

* *Whilst for some of the comparisons the fold change value would suggest differential expression, those genes were not considered as DE since they failed to meet the fdr requisite (<0.05). For a list of all genes considered as DE in every comparison, see Supplementary Tables 4, 5, 7*.

#### Early Parasite Adaptation to Intracellular Lifestyle Seems to Modulate Host TCA Cycle Metabolic Machinery and Down Regulates Mechanisms Involved in Endothelium Protection

When *B. besnoiti* infected BAEC at 12 hpiwere compared to uninfected BAEC, only 57 DEGs were identified, of which 15 were up-regulated and 42 down-regulated (Supplementary Table 4). This low number of DEGs may be due to the low infection rate achieved with the reference strain employed (BbSpain1 is a low invader and low prolific isolate; Frey et al., 2016), since only up to 20% of the cells were infected (data not shown) biasing the results. Moreover, it has been reported that parasites modulate host cell gene expression only when they have already adapted to the intracellular lifestyle inside the parasitophorous vacuole (Hakimi et al., 2017). Thus, enriched GO terms under the three domains assayed (biological process, molecular function or cellular component) (PANTHER overrepresentation test, Complete GO) were not found. However, two Panther Pathways were significantly enriched: Succinate—proprionate conversion and methylmalonyl pathway. KEGG pathways analysis showed an enrichment of “Carbon metabolism” pathway (bta01200). These results indicate that genes in the tricarboxylic acid cycle (TCA) pathway are regulated in infected BAECThis glucose deprivation from the parasitized host cell might arrest cell cycle progression in conditions of starvation (Kalucka et al., 2015). It is known that the closely related apicomplexan *Toxoplasma gondii* scavenges carbon sources (glucose and glutamine) from the host cell to fuel its own TCA cycle (Blader and Koshy, 2014; Jacot et al., 2016), and is able to induce a G2/M host cell cycle arrest in primary bovine endothelial cells (BUVEC) (Velásquez et al., 2019). Previous results in *B. besnoiti* have already shown that the TCA pathway is a key energy pathway for tachyzoites according to Fernández-García et al. (2013), who compared the tachyzoite and bradyzoite stage using proteomic approaches and showed that most of the identified proteins were glycolytic enzymes. Taubert et al. (2016) also showed that the parasite triggered an up-regulation of glycolysis and glutaminolysis *in vitro* using pathway-specific inhibitors.

**Figure 1:**
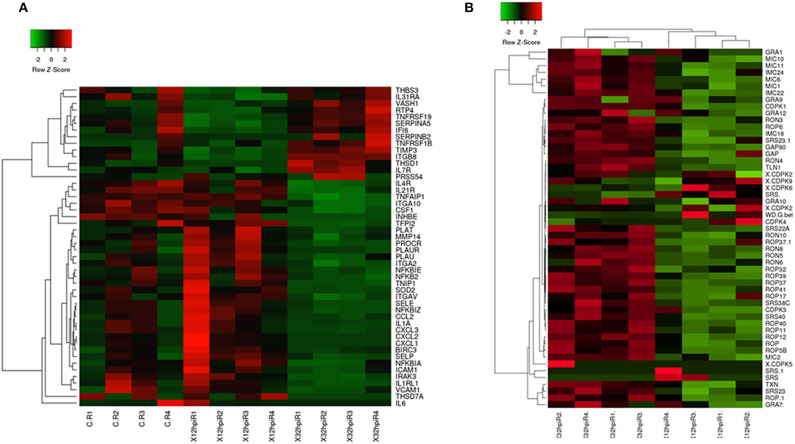
Heatmaps of a selection of *Bos taurus* and *Besnoitia besnoiti* differentially expressed genes. **(A)** Heatmap showing patterns of expression (normalized reads) in a selection of *B. taurus* differentially expressed genes implicated in relevant pathways mentioned in the manuscript (e.g., endothelial activation, leukocyte recruitment, proinflammatory phenotypes, extracellular matrix organization). A complete list of *B. taurus* genes that are included in the figure is available in Supplementary Table 11. **(B)** Heatmap displaying the transcriptional abundance of a selection of *B. besnoiti* genes. A complete list of *B. besnoiti* genes that are included in this figure is available in Supplementary Table 12. The heatmaps were generated using Heatmapper (http://www2.heatmapper.ca/). The genes were clustered using the Pearson computing distance method.

Genes involved in the protection of endothelial cells (ECs) were shown to be downregulated at 12 hpi (Table 1, Supplementary Table 4, Figure 1A). Among these, we found genes associated to normal EC function involved in the response to oxidative stress, such as NOX5 and SOD3, protease inhibitors (SERPIN5) and endothelial specific molecules (EPAS1, ECM2). NOX5 is responsible for the generation of reactive oxygen species (ROS) in response to several stimuli and its expression is accompanied by an increase in EC proliferation and promotes their organization into capillary networks (Fulton, 2009). SOD3 is a secreted enzyme responsible for the redox balance in specific tissues, including ECs, preventing oxidative damage and preserving nitric oxide (NO) availability (Fukai and Ushio-Fukai, 2011). SERPIN5 is a glycoprotein that can inhibit serine proteases, including plasminogen activators (Azhar et al., 2013). Our results are thus indicative of endothelial dysfunction in *B. besnoiti* infected BAEC. Additionally, the highest fold change observed for DEG in infected BAEC at 12 hpi corresponded to ADAMTS1 (Supplementary Table 4), a gene belonging to a family named as a disintegrin and metalloproteinase with thrombospondin motifs (ADAMTS). The regulation of ADAMTS1 may suggest an effect on extracellular matrix organization (ECM) as a response to tachyzoite invasion. This family of protease enzymes has been shown to be capable of degrading essentially all components of the ECM to facilitate tissue remodeling (Dancevic et al., 2016). In particular, ADAMTS1 may contribute to the dissemination of *Toxoplasma* infected leukocytes into immune-privileged sites (Seipel et al., 2010) and its gene expression is also modulated upon *N. caninum* infection of trophoblast cells (Horcajo et al., 2017).

**Figure 2:**
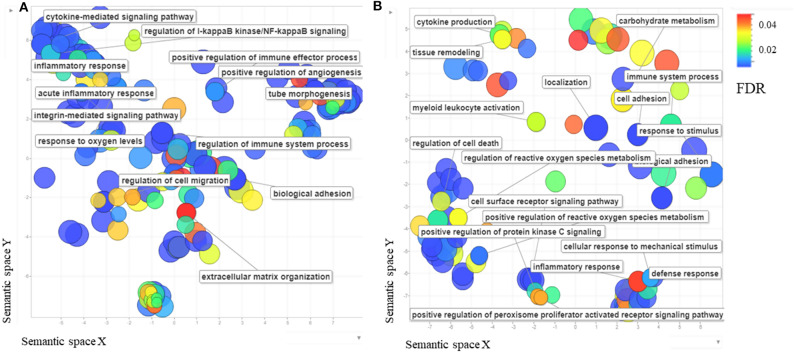
**(A)** The scatterplot shows the cluster representative genes of enriched biological process Gene Ontology (GO) terms among differentially expressed *Bos taurus* genes in *Besnoitia besnoiti*-infected BAEC cells at 32 hpi vs. non-infected BAEC summarized using REVIGO (Supek et al., 2011). **(B)** The scatterplot shows the cluster representatives of enriched biological process GO terms among differentially expressed *Bos taurus* genes in *Besnoitia besnoiti*-infected BAEC cells at 32 hpi vs. *Besnoitia besnoiti*-infected BAEC cells at 12 hpi summarized using REVIGO. The axes, semantic spaces x and y, mean that more semantically similar GO terms are closer in the plot.

396 genes were DEG when infected BAEC at 32 hpi were compared to uninfected cells, of which 161 were upregulated at 32 hpi. Among those genes upregulated at 32 hpi, the highest fold change observed was for MT1A, and ADAMTS1 (Supplementary Table 5). When this subset of DEG were Analyzed (Supplementary Table 5), terms related to both innate and adaptive immune responses, as well as responses to wounding, were found (Supplementary Table 6, Figure 2A). Besides, KEGG pathways such as “TNF-α signaling pathway (bta 04510)” or “TGF-β signaling pathway (bta04350)” were present (Supplementary Table 6). These results correlate with the activation of ECs described at 12 hpi, but it seems that the proinflammatory response is in a later stage as a consequence of the initial injury of ECs. A remodeling of ECM and angiogenesis regulation are present, evidenced by the expression of genes coding for thrombospondin precursors, fibronectin, ADAMTS, integrin β7 (ITGB7) and vascular endothelial growth factor A (VEGFA), among others.

#### Type II Endothelial Activation Is Progressively Induced by *B. besnoiti* Tachyzoites in BAEC Along the Lytic Cycle

Endothelial cell dysfunction was corroborated when infected BAEC at 32 hpi and infected BAEC at 12 hpi were compared, since the highest number of DEG (n = 445) were found, compared to the pair wise comparisons that included infected BAEC at 12 hpi or 32 hpi*vs*. non-infected BAEC (Supplementary Table 7, Figure 1A), together with relevant enriched pathways. Of this subset of DEGs, 249 DEGs were up-regulated and 196 DEGs were down-regulated in infected BAEC at 32 hpi (Supplementary Table 7).

Upon *B. besnoiti* infection, BAEC showed features compatible with a type II activation (e.g., in response to pathogen stimulation or to cytokines such as IL-1, TNFα and IFNγ) (Mai et al., 2013; Gimbrone and García-Cardeña, 2016) (Figure 2B). Endothelial cell activation comprises a sequence of events involved in many pathological processes: (i) loss of vascular integrity; (ii) upregulation of leukocyte adhesion molecules that leads to leukocyte recruitment, (iii) coagulation including platelet aggregation, coagula formation and reduced fibrinolytic potential (Gimbrone and García-Cardeña, 2016). DEGs related with EC activation were found overexpressed at both pi time points. Genes coding for proteins involved in early activation steps were overexpressed at 12 hpi, whereas DEGs related with a later stage of activation were found overexpressed at 32 hpi.

Loss of vascular integrity was reflected by several genes and GO terms (Supplementary Table 8, Figure 2B) associated with ECM remodeling found to be regulated upon infection. We identified matrix metalloproteinases (such as MMP14) upregulated at 12 hpi, which are able to degrade ECM for tissue remodeling (Parks et al., 2004; Nagase et al., 2006). Previous studies in the closely related parasites, *T. gondii* and *N. caninum*, have shown a regulation of other MMPs, which may be important for crossing biological barriers and intra-organic dissemination (Horcajo et al., 2017). Other relevant proteins for ECM remodeling included the upregulation of ADAMTS as previously mentioned. This loss of integrity suggested by these *in vitro* studies may be responsible for changes in vascular permeability that are thought to be responsible for oedemas and hemorrhages during the acute phase of bovine besnoitiosis (Álvarez-García et al., 2014). Other ECM related genes were also modulated: Integrin alpha 2, integrin alpha V and integrin V10 were upregulated at 12 hpi whilst fibronectin (FN1) collagen-alpha and integrin beta 8 were found to be upregulated at 32 hpi. In fact, integrin-mediated signaling was found to be enriched using Panther tools (Supplementary Table 8). In ECs, integrins mediate the blood flow shear stress response (Muller et al., 1997; Shyy and Chien, 2002). Finally, another DEG involved in the integrity of the endothelial barrier was claudin-1, a gene coding for a protein present in the host cell tight-junctions (Morita et al., 1999), which was also upregulated at 32 hpi. The overexpression of claudin-1 may reflect a wound-healing response initiated after the initial injury and activation of endothelial cells upon parasite invasion, as it has been described for claudin-1 in other systems (Shi et al., 2018).

**Figure 3:**
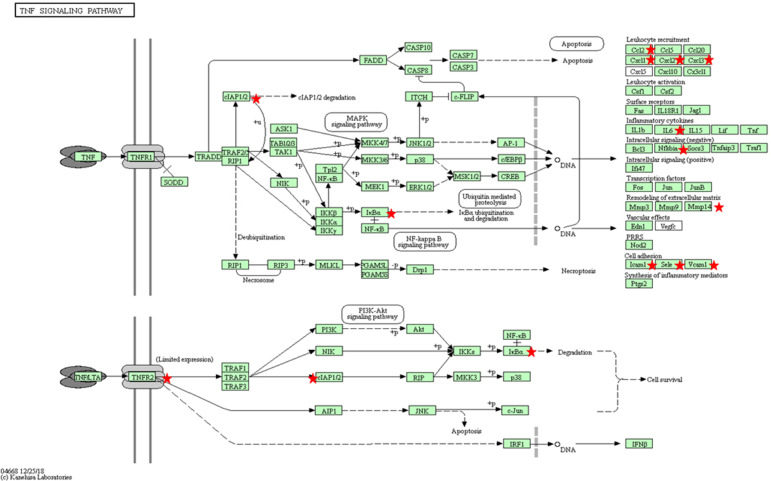
KEGG pathway for TNFα (bta04668) with the annotated DEGs in our results between *Besnoitia besnoiti*-infected BAEC cells at 32 hpi vs. *Besnoitia besnoiti*-infected BAEC cells at 12 hpi (Kanehisa, 2019). Red stars represent DEGs genes in the pathway. Leukocyte recruitment: CCL2, CXCL1, CXCL2, CXCL3; Inflammatory cytokines: IL-6; Intracellular signaling (negative): NFKBIA; IκBa; Remodeling of extracellular matrix: MMP14; Cell adhesion: ICAM1, VCAM1, SELE.

Another key step in the endothelium activation is the expression of surface markers, which may lead to an increase in adhesion of leukocytes. We found as DEGs canonical markers of endothelial activation such as selections (SELE and SELP), as well as VCAM and ICAM1, upregulated in infected BAEC at 12 hpi when compared to 32 hpi (Supplementary Table 7). E-selectin expression depends on P-selectin upon pro-inflammatory modulators such as IL-1 (Leeuwenberg et al., 1992), which was also upregulated at 12 hpi. VCAM-1 has been shown to present a sustained expression pattern in cultured endothelial cells upon cytokine stimulation (Choi et al., 2018), and shows selective adhesive properties for mononuclear leukocytes and lymphocytes (Osborn et al., 1989). Activated ECs have an intrinsic capacity to secrete chemokines and cytokines such as IL-1, IL-6, CCL2, or CXCL-1, as they form part of the innate immune system and work as a first danger signal sensor (Mai et al., 2013). The secretion of these signaling molecules is responsible for the generation of localized signaling loops which may contribute to the increase in vascular permeability that has been hypothesized to occur in acute bovine besnoitiosis and is induced by other bovine pathogens responsible for endothelial dysfunction such as bluetongue virus (BTV) (DeMaula et al., 2002). The different chemokines secreted by ECs are able to selectively attract different subpopulations of leukocytes, such as monocytes (CCL2 also known as MCP1, Deshmane et al., 2009), neutrophils (CXCL1, Scapini et al., 2004) or eosinophils (CCL24, also known as eotaxin2, Tsai et al., 2016). These findings are supported by TNF-α- KEGG pathway, genes of which were found to be overrepresented in the set of DEGs upon *B. besnoiti* infection in the 32 vs. 12 hpi comparison, being overexpressed at 12 hpi (Supplementary Table 8, Figure 3). TNF-α is a key proinflammatory cytokine under the control of the NF-kβ transcription factor together with other potent upregulated inflammatory cytokines, such as IL-6 and IL-1A. In healthy barrier cells (such as epithelial and endothelial cells), a steady expression of IL-1A has been demonstrated (Garlanda and Mantovani, 2013; Garlanda et al., 2013), but its expression can be induced by canonical proinflammatory mediators (Weber et al., 2010; Rider et al., 2012). This cytokine is responsible for the establishment of an inflammatory loop, where stressed or damaged cells produce an IL-1A-dependent activation of chemokines that recruit leukocytes. Also, IL-1A is able to modulate the expression of other proinflammatory cytokines, such as IL-1B, at a transcriptional level, which may amplify the inflammatory loop (Di Paolo and Shayakhmetov, 2016). Besides, *in vivo* studies performed in mice have shown that interactions between TNF-α and IL-6 exacerbate oxidative stress and reduce the phosphorylation of endothelial nitric oxide synthase (eNOS), leading to an endothelial dysfunction (Lee et al., 2017). In this context, the pleiotropic transcription factor NF-kB seems to play a pivotal role since it coordinates the expression of additional effector proteins that appeared as DEGs, such as E-Selectin (SELE), VCAM1, IL-6 and CCL-2, conferring a locally coordinated proinflammatory phenotype. Moreover, several inhibitors and NFkB2 genes were upregulated at 12 hpi indicating that this pathway might be modulated upon parasite-infection.

A key gene with an antioxidant effect is metallothionein 1A (MT1A), which was found to be upregulated in infected BAEC at 32 hpi and may reflect an attempt to counteract the inflammatory phenotype induced by the parasite proliferation. Also, genes in the KEGG PPAR peroxisome pathway, which provides vascular protection upon oxidative stress (Mukohda et al., 2015) were found to be modulated upon *B. besnoiti* infection (Supplementary Table 8).

Our results partially agree with the findings reported by Maksimov et al. (2016), where the transcription of a small panel of adhesion molecules, chemokines, and regulatory molecules (*n* = 12) was studied upon *B. besnoiti* infection by qPCR up to 48 hpi. However, in their study a few statistically significant differences were observed, i.e., up regulation of ICAM at 24 hpi, P-SELE at 6 and 12 hpi, chemokines (CXCL-1, CXCL-8, and CCL5) between 6 and 48 hpi and of IL-6 and COX-2 at 48 hpi. In contrast, the relevance of other genes such as VCAM was not proved. These differences in their results compared with our observations might be explained by the different *in vitro* model employed, including the host cell (BUVEC), *B. besnoiti* isolate (Bb1Evora04) and the MOI.

Coagulation, exemplified by fibrinolysis pathways, has been also found to be modulated by the parasite in the present work, with the upregulation of several molecules with fibrinolytic properties such as plasminogen activator, urokinase receptor (PLAUR) and plasminogen activator, tissue type (PLAT) at 12 hpi. In addition, fibronectin and thrombospondin are adhesins which favor the platelet adhesion that were also upregulated at 32 hpi.

These pathways induced are in accordance with the microscopic lesions found *in vivo*, such as vasculitis and thrombosis, in skin and circulatory system (Pols, 1960; McCully et al., 1966; Basson et al., 1970).

#### BAEC Infected With *B. besnoiti* Show a Profibrotic Phenotype

**Figure 4:**
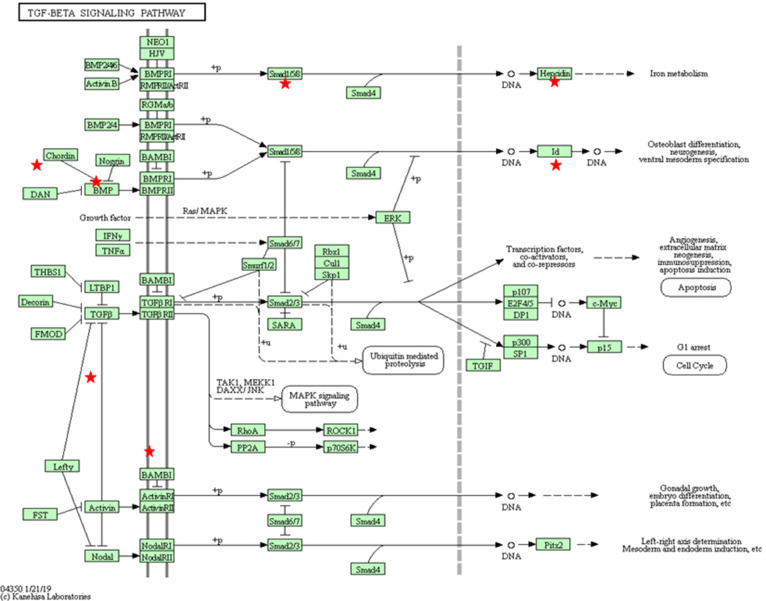
KEGG pathway for TGF-β (bta04350) with the annotated DEGs in our results between *Besnoitia besnoiti*-infected BAEC cells at 32 hpi vs. non-infected BAEC cells (Kanehisa, 2019). Red stars represent DEGs in the pathway: Mothers against decapentaplegic homolog 9 (SMAD9); Activin A receptor type 1C (ACVR1C); Inhibin beta E subunit (INHBE); Inhibitor of DNA binding 1 (ID1); ID2; ID3; latent transforming growth factor beta binding protein 1 (LTBP1); thrombospondin 1 (THBS1); v-myc avian myelocytomatosis viral oncogene homolog (MYC).

Fibrosis plays a crucial role in the pathogenesis of scleroderma, which is characteristic of chronic besnoitiosis. Fibrosis includes the following sequential steps: inflammation, myofibroblast differentiation, ECM accumulation and angiogenesis (Sahin and Wasmuth, 2013). Several fibrosis markers were upregulated at 12 hpi, including chemokines responsible for the recruitment of monocytes and macrophages, such as CCL2, IL-6, and IL-1A and mediators of macrophage differentiation such as macrophage colony stimulating factor 1 (MCSF-1). This macrophage recruitment could explain the predominance of activated macrophages among the leukocyte populations in the inflammatory foci around tissue cysts in chronic besnoitiosis (Frey et al., 2013). The interaction of CCL2 with its receptor CCR2 has been implicated in many fibrotic processes (Sahin and Wasmuth, 2013). Besides its role as a monocyte chemoattractant, this molecule is able to mediate the activation of fibroblasts to produce TGF-β and thus, to stimulate collagen synthesis leading to the deposit of collagen and fibroid tissue (Gharaee-Kermani et al., 1996). Indeed, macrophages are major sources of growth factors that induce ECM deposition. Besides, the KEGG pathway for TGF-β (Supplementary Table 6, Figure 4) was enriched in our subset of DEGs. Since scleroderma is commonly associated with the chronic stage of bovine besnoitiosis, CCL2 may play a key role in the pathogenesis of bovine besnoitiosis. Other relevant DEG were proheparin-binding EGF-like growth factor, which is involved in macrophage mediated cellular proliferation and has mitogenic properties for fibroblasts (Jin et al., 2002), and integrins that allow the anchoring of ECs to components of ECM, such as fibronectin or collagen, and regulating the TGF-β pathway (Henderson et al., 2013).

**Figure 5:**
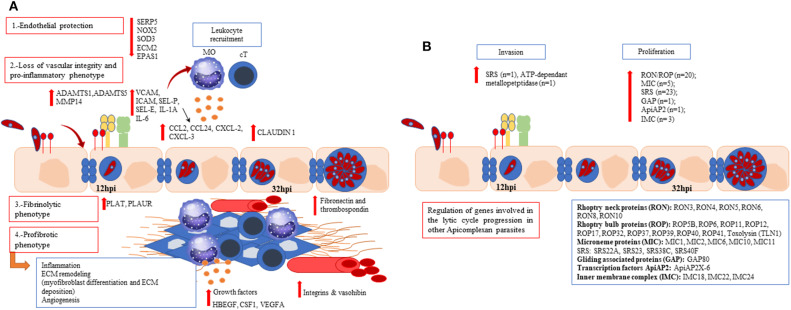
Graphical abstract with the main findings of up- and down-regulated host **(A)** and parasite **(B)** pathways/genes during an *in vitro Besnoitia besnoiti* infection in primary bovine endothelial cells. **(A)** A progressive endothelial dysfunction along the parasite lytic cycle is evidenced. *Bos taurus* differentially expressed genes are associated with reduced endothelial protection (SOD3, NOX5, ECM2, EPAS1, SERPIN5); a proinflammatory, fibrinolytic and profibrotic phenotype represented by cytokines (IL-1A, IL-6), chemokines (CXCL-2, CXCL-3, CCL-2, CCL24), surface adhesion markers (ICAM-1, VCAM-1, SELE, SELP), genes involved in the coagulation cascade (PLAT, PLAUR) and growth factors (HBEGF, CSF-1; VEGFA) for both post-infection times assayed. **(B)** Genes coding for surface proteins (SRS), microneme proteins (MIC), AP-2 transcription factors, rhoptry kinases (ROP), rhoptry neck proteins (RON), and genes coding for proteins such as apical membrane antigen-1 (AMA-1) and gliding associated proteins (GAP) were regulated along *B. besnoiti* lytic cycle. Mo, macrophages; cT, cytotoxic T lymphocytes.

Angiogenesis was represented by several DEGs. Thus, DEGs involved in both early (growth factors and matrix metalloproteinases) (Brigstock, 2002) and late steps (integrins and vasohibin) evidenced the regulation of this multistep process. The activation of ECs in response to angiogenic factors can lead to a degradation of the endothelial barrier by the secretion of extracellular proteinases (such as MMP or ADAMTS). This degradation lead to a branch point in the vessel wall, with the synthesis of integrins that allow the endothelial cells to migrate toward the angiogenic stimulus. Afterwards, ECs are re-organized to form tubules which are cleaved and interconnected to form a network. Our results show a modulation of several angiogenic molecules (e.g., integrins, growth factors, ADAMTS1, MMP14), including an upregulation of vasohibin-1 at 32 hpi, that is specifically expressed by ECs (Kosaka et al., 2013). Figure 5A shows a graphical abstract of the regulation of host pathways/genes during infection.

#### qPCR Validation of Bos Taurus Genes

A total of 17 *B. Taurus* DEGs (CCL2, CCL24, CXCL2, CXCL3, IL-6, IL-1A, ICAM-1, VCAM-1, SELE, PLAT, PLAUR, NFKB2, thrombospondin1 (THBS1), MT1A, ADAMTS1, FN1, ADAMTS4) were selected for qPCR validation due to their roles in DEG-enriched pathways and/or relevant signaling routes (endothelial activation, leukocyte recruitment, fibrosis and ECM remodeling). Quantitative real-time PCR results showed a similar profile compared with the RNA-Seq results, with a similar significance and direction of fold change in >85% of the validations (Supplementary Figure 2). Even though these genes (SELE, IL-6, IL-1A, CCL2, CCL24, CXCL2, CXCL3) did not appear as DEGs in infected BAEC at 12 hpi vs. non infected BAEC.

### *Besnoitia besnoiti* Transcriptome

#### *Besnoitia besnoiti* Tachyzoites Display a Similar Gene Expression Profile of Genes Involved in the Parasite Lytic Cycle at Early and Late Stage Infection

Our results showed that several *B. besnoiti* genes exclusively present in apicomplexan parasites were expressed at both infection time points, such as dense granule proteins (GRAs) or calcium-dependent kinases (CDPKs), without being differentially expressed when both post-infection times were compared (Figure 1B).

Similarly, in *N. caninum*, GRAs, such as NcGRA7, NcNTPase (Pastor-Fernández et al., 2016) showed higher expression after early invasion and at egress. This was also described for several *T. gondii* GRA proteins (Radke et al., 2005) that were shown to be relevant in invasion of the host cell, maturation of the parasitophorous vacuole and egress (Blader et al., 2015).

The present work identified at least eight putative CDPKs in the *B. besnoiti* genome (Supplementary Table 9) and expressed on the tachyzoite stage. CDPKs are kinases absent in the mammalian host and have therefore garnered significant interest as potential drug targets, allowing the development of specific bumped kinase inhibitors (BKIs), which have been shown to be effective both *in vitro* and *in vivo* against a wide range of apicomplexan parasites (reviewed by Van Voorhis et al., 2017) including *B. besnoiti* (Jiménez-Meléndez et al., 2017). CDPK1 is required for microneme secretion in *T. gondii* and is thus essential for parasite invasion (Lourido et al., 2010, 2012), while CDPK3 is involved in the K^+^-mediated egress pathway (McCoy et al., 2012). Besides, BKI treatment of *B. besnoiti* tachyzoites impaired parasite invasion and proliferation (Jiménez-Meléndez et al., 2017). Also, genes coding for aspartyl proteases (ASP), enzymes important in the cleavage of proteins, have become of increasing interest in the drug design against *T. gondii* (Dogga et al., 2017) and have been detected in the present work.

However, despite the similar expression profile, we successfully identified 105 DEGs when the two infection time points were compared, and only eight genes were upregulated at 12 hpi compared to 32 hpi (Supplementary Table 10; Figure 1B). We were not able to identify significantly enriched GO terms or pathways due to the high proportion of hypothetical proteins (~40%) in the DEGs.

Among those DEGs, many of which are involved in the lytic cycle in other Toxoplasmatinae parasites, we found genes coding for surface proteins, microneme proteins, AP-2 transcription factors, rhoptry kinases, rhoptry neck proteins, and genes coding for proteins such as apical membrane antigen-1 (AMA-1) and gliding associated proteins (GAP) (Supplementary Table 10), that were regulated along the lytic cycle. Those proteins had not been previously identified by proteomics approaches due to the absence of the whole genome sequence (Fernández-García et al., 2013; García-Lunar et al., 2013, 2014).

SAG-related sequence (SRS) proteins mediate low-affinity parasite interactions with the host-cell membrane for parasite adhesion. Our results have shown that 22 genes encoding SRS proteins were upregulated in tachyzoites at 32 hpi, whilst only one was upregulated at 12 hpi (Supplementary Table 10). One gene encoding a glideosome proteins -GAP80-, which is essential for gliding motility, was upregulated at 32 hpi (Supplementary Table 10).

After the initial recognition of the host cell mediated by SRS proteins, a tighter interaction mediated by microneme proteins leads to the formation of the moving junction (MJ). This MJ is established between the microneme protein AMA1 and proteins from the neck of the rhoptries (RON proteins), which are secreted before kinases from the bulb of the rhoptries (ROPs) (Blader et al., 2015). Our results have shown a differential expression profile for RON3, RON4, RON5, RON6, RON8 and RON10, all of them upregulated at 32 hpi (Supplementary Table 10). This finding may indicate that at 32 hpi, tachyzoites are already preparing the invasion machinery for the entry in new host cells after egress. In *T. gondii*, it has been shown that RON8 associates to the MJ complex for proper invasion (Straub et al., 2009). On the other hand, RON4 and RON5 have a structural function in the architecture of the complex (Besteiro et al., 2009, 2011). Additionally, RON6 forms a complex with other RON proteins, such as RON2, RON4 and RON8, and is associated with AMA1 (Muniz-Feliciano et al., 2013). RON10 forms a complex with RON9 in *T. gondii* but it is not detected in the MJ during invasion and it is not associated with the core complex formed by RON2/4/5/8 and AMA1. Finally, it was reported that RON3 was also DE, but in *T. gondii* it has an unknown function; in *P. falciparum*, RON3 has been shown to play an important role in merozoite invasion intro erythrocytes (Zhao et al., 2014).

Among the rhoptry bulb proteins, several ROPs have been shown to be DEG, such as ROP5B, ROP17, ROP40, which were overexpressed at 32 hpi (Supplementary Table 10). In *T. gondii*, several ROPs have been described as effectors that hijack host immune responses (Hakimi et al., 2017). For instance, ROP5, ROP17, and ROP18 forming ROP5/17/18 complex function as virulence factors to prevent the accumulation of immune related GTPases (iRG) at the parasitophorous vacuole (PV) (Saeij et al., 2006). So far, the phosphorylation of iRGs has not been described in *B. besnoiti* as a strategy to subvert the host-immune responses, but since mice are not a natural host for *B. besnoiti* and iRGs, as well as TLR11 and TLR12, are absent from the *Bos taurus* genome this may not be as relevant as described for *T. gondii* infections in mice. Further studies should address whether these ROPs represent putative virulence factors in *B. besnoiti*.

Of the genes that encode for inner-membrane complex (IMC) proteins, that play a crucial role in parasite replication, we found 3 DEG: IMC18, IMC22 and IMC24, all of them upregulated at 32 hpi (Supplementary Table 10).

Among transcription factors, the AP2 family have members which coordinates many developmental processes in *T. gondii* (Walker et al., 2013). However, our results have shown only AP2X6 transcription factor as DEG (Supplementary Table 10), whose function in other coccidian's has not been fully elucidated yet. Figure 5B shows a graphical abstract of the regulation of parasite genes during infection.

#### qPCR Validation of *Besnoitia besnoiti* Genes

Eleven *B. besnoiti* genes were selected for qPCR validation according to their implications in key processes in other apicomplexan parasites (MIC2, MIC11, GAP80, ROP5B, ROP17, ROP40, GRA7, GRA10, SRS22A, PEP, SRS). The qPCR results showed a similar profile to the RNA-Seq results, with a similar significance and direction of fold change in >90% of the validations (Supplementary Figure 3).

## Concluding Remarks

This work represents the first transcriptomics approach by means of RNA-Seq to study *in vitro* host-pathogen interaction between *B. besnoiti* and target cells during the acute infection. The different steps of ECs activation were progressively induced, a process associated to vascular injury and correlated with microscopic lesions described in tissues from naturally and experimentally infected cattle. Rapid progression of a proinflammatory, procoagulant and profibrotic state was triggered upon parasite invasion.

Additionally, we have identified for the first time many orthologous genes from other Toxoplasmatinae parasites, some of which are upregulated during proliferation (e.g., MIC2, MIC11, ROP40, RON2, GAP80, SRS). This work may help to search for potential drug and vaccine targets and promising markers of disease and prognosis (such as IL-6, IL-1A, CCL2, that showed some of the highest fold change values), that should be further studied *in vivo*.

## Data Availability Statement

The data that support the findings of this study have been deposited in Gene Expression Omnibus (GEO) repository at https://www.ncbi.nlm.nih.gov/geo, with reference number GSE139306.

## Author Contributions

GA-G and AH conceived the study and participated in its design. AJ-M performed *in vitro* infection experiments, functional enrichment and network analysis and wrote the manuscript, with interpretation of results and discussion inputs from CR, GR, AH, and GA-G. GR performed computational analysis of RNA-Seq data. AJ-M designed and performed RT-PCR analyses. All authors read and approved the final manuscript.

## Conflict of Interest

The authors declare that the research was conducted in the absence of any commercial or financial relationships that could be construed as a potential conflict of interest.

## Supplementary Material

Supplementary Figure 1 2D mdsPlot of top genes from the samples analyzed in the present work.

Supplementary Figure 2 RT-PCR validation results for *Bos taurus* differentially expressed genes in every comparison performed. **(A)** RT-PCR validation results for 12 hpi vs. C-. Positive fold-change values indicate gene upregulation in infected BAEC at 12 hpi. **(B)** RT-PCR validation results for 32 hpi vs. C-. Positive fold-change values indicate gene upregulation in infected BAEC at 32 hpi. **(C)** RT-PCR validation results for 32 vs. 12 hpi comparisonPositive fold-change values indicate gene upregulation in infected BAEC at 32 hpi.

Supplementary Figure 3 RT-PCR validation results for *Besnoitia besnoiti* differentially expressed genes. Positive fold-change values indicate gene upregulation in infected BAEC at 32 hpi.

Supplementary Table 1 Primers used for RT-PCR validation of *Bos taurus* genes.

Supplementary Table 2 Primers used for RT-PCR validation of *Besnoitia besnoiti* genes.

Supplementary Table 3 Mapped reads against *Bos taurus* and *Besnoitia besnoiti* in all samples analyzed in the present work.

Supplementary Table 4 Differentially expressed genes (DEGs) mapped to *Bos taurus* at 12 hpi vs. C- comparison. Positive fold-change values indicate gene upregulation in infected BAEC at 12 hpi.

Supplementary Table 5 Differentially expressed genes (DEGs) mapped to *Bos taurus* at 32hpi vs. C- comparison. Positive fold-change values indicate gene upregulation in infected BAEC at 32 hpi.

Supplementary Table 6 Results from the Gene Ontology (GO) analysis in the category for biological process complete (bp complete) at 32 hpi vs. C- comparison.

Supplementary Table 7 Differentially expressed genes (DEGs) mapped to *Bos taurus* at 32 hpi vs. 12 hpi comparison. Positive fold-change values indicate gene upregulation in infected BAEC at 32 hpi.

Supplementary Table 8 Results from the Gene Ontology (GO) analysis in the category for biological process complete (bp complete) at 32 vs. 12 hpi.

Supplementary Table 9 Expression profile of selected families of *B. besnoiti* genes (dense granules, GRA; calcium dependent protein kinases, CDPKs; AP2 transcription factors and Aspartyl proteases, ASP).

Supplementary Table 10 Differentially Expressed Genes (DEGs) mapped to *Besnoitia besnoiti* at 32 vs. 12 hpi comparison. Positive fold-change values indicate gene upregulation in infected BAEC at 32 hpi.

Supplementary Table 11 Complete list of the abundance of the expression data from *B. taurus* genes included in Figure 1A.

Supplementary Table 12 Complete list of the abundance of the expression data from *B. besnoiti* genes included in Figure 1B.
