# Supplementary figures and images for: RNA-Seq Analyses Reveal That Endothelial Activation and Fibrosis Are Induced Early and Progressively by Besnoitia besnoiti Host Cell Invasion and Proliferation
Source: Front Cell Infect Microbiol. 2020 May 15;10:218. doi: 10.3389/fcimb.2020.00218 (PMC7242738; doi:10.3389/fcimb.2020.00218)

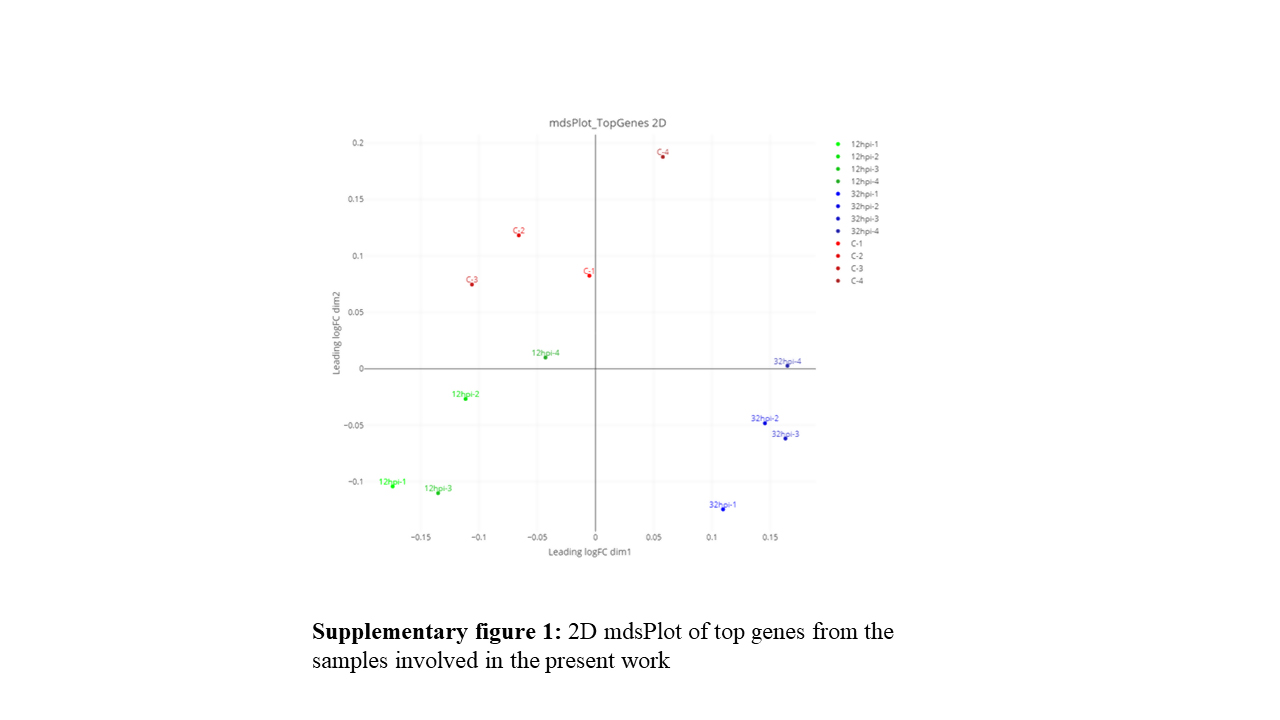

Supplement: Supplementary Figure 1 — 2D mdsPlot of top genes from the samples analyzed in the present work. [file Image_1.JPEG]

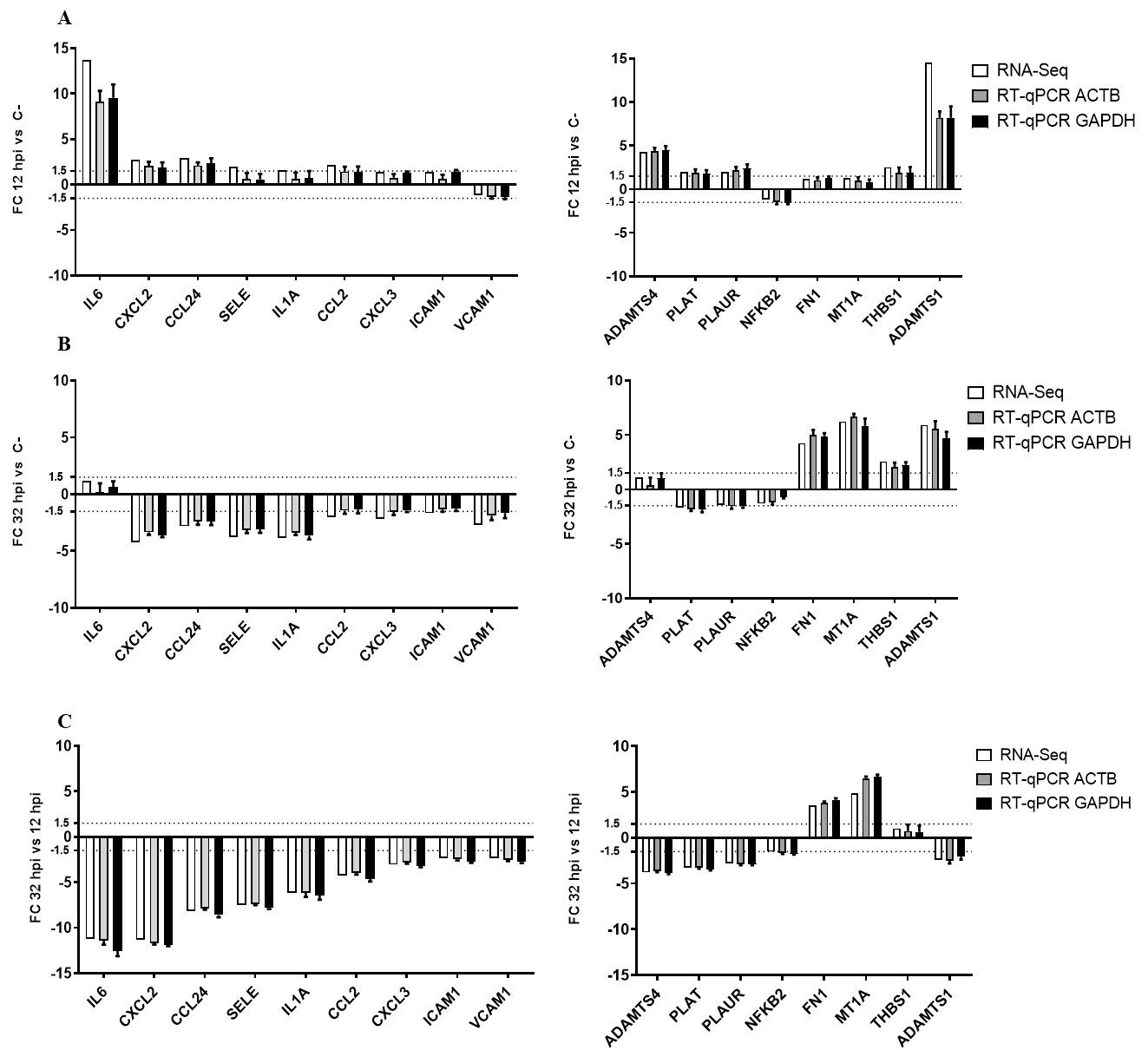

Supplement: Supplementary Figure 2 — RT-PCR validation results for Bos taurus differentially expressed genes in every comparison performed. (A) RT-PCR validation results for 12 hpi vs. C-. Positive fold-change values indicate gene upregulation in infected BAEC at 12 hpi. (B) RT-PCR validation results for 32 hpi vs. C-. Positive fold-change values indicate gene upregulation in infected BAEC at 32 hpi. (C) RT-PCR validation results for 32 vs. 12 hpi comparisonPositive fold-change values indicate gene upregulation in infected BAEC at 32 hpi. [file Image_2.JPEG]

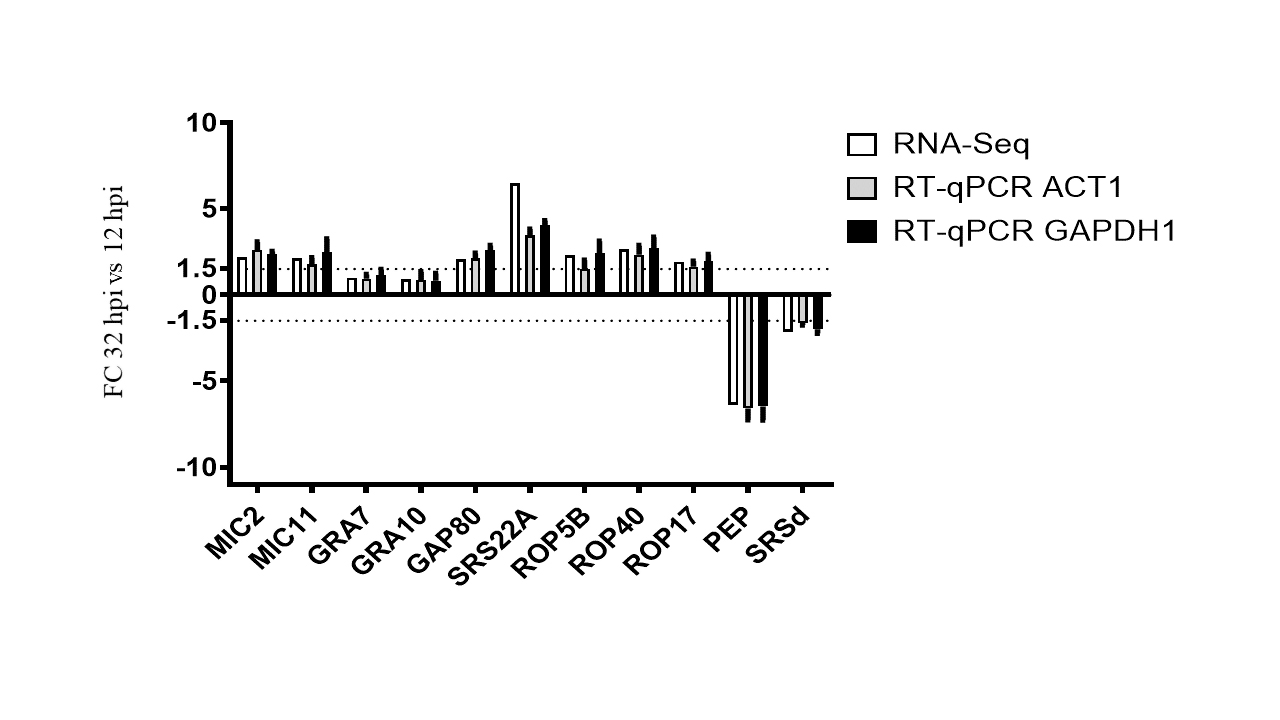

Supplement: Supplementary Figure 3 — RT-PCR validation results for Besnoitia besnoiti differentially expressed genes. Positive fold-change values indicate gene upregulation in infected BAEC at 32 hpi. [file Image_3.JPEG]
