# Supplementary material for: RNA-Seq Analyses Reveal That Endothelial Activation and Fibrosis Are Induced Early and Progressively by Besnoitia besnoiti Host Cell Invasion and Proliferation
Source: Front Cell Infect Microbiol. 2020 May 15;10:218. doi: 10.3389/fcimb.2020.00218 (PMC7242738; doi:10.3389/fcimb.2020.00218)
Supplement: Supplementary Table 1 — Primers used for RT-PCR validation of Bos taurus genes. [file Table_1.DOC]

| **Gene name** | **Gene Symbol** | **Ensembl database ID** | **Primer sequence (5`-3`)** | **Reference** |
| --- | --- | --- | --- | --- |
| Chemokine (C-X-C motif) ligand 2 | CXCL2 | ENSBTAG00000037558 | Fw: TGGTCAGGAAGTGTGTCTCAA | Magee et al., 2012 |
|  |  |  | Rv: TCAGTTGGCACTAGCCTTGTT |  |
| Chemokine (C-X-C motif) ligand 3 | CXCL3 | ENSBTAG00000037778 | Fw: GCC ATT GCC TGC AAA CTT | Gartner et al., 2016 |
|  |  |  | Rv: TGC TGC CCT TGT TTA GCA |  |
| Chemokine (C-C motif) ligand 2 (MCP-1) | CCL2 | ENSBTAG00000037811 | Fw: TGCAGACCCCAAGCAGAAAT | Sakumoto et al., 2017 |
|  |  |  | Rv: AGAGGGCAGTTAGGGAAAGC |  |
| Chemokine (C-C motif) ligand 24 | CCL24 | ENSBTAG00000026275 | Fwd: TAGAGGGCTCTTGGTCACA | Negrón-Pérez et al., 2017 |
|  |  |  | Rv: GTCCTCCAGGTCCATTCATTAC |  |
| Interleukin 1, alpha | IL1A | ENSBTAG00000010349 | Fw: CTCTCTCAATCAGAAGTCCTTCTATG | Ito and Kodama, 1996 |
|  |  |  | Rv: CATGTCAAATTTCACTGCCTCCTCC |  |
| Interleukin 6 | IL6 | ENSBTAG00000001491 | Fw: CTGGGTTCAATCAGGCGATT | Arranz-Solís et al., 2016 |
|  |  |  | Rv: GGATCTGGATCAGTGTTCTGA |  |
| Intercellular Adhesion Molecule 1 | ICAM-1 | ENSBTAG00000010303 | Fw: AGACCTATGTCCTGCCATCG | Jiménez-Pelayo et al., 2019 |
|  |  |  | Rv: GGTGCCCTCCTCATTTTCCT |  |
| vascular cell adhesion molecule 1 | VCAM-1 | ENSBTAG00000007773 | Fw: GAACTGGAAGTCTACATCTC | Jiménez-Pelayo et al., 2019 |
|  |  |  | Rv: CAGAGAATCCGTGGAGCTGG |  |
| E-Selectin | SELE | ENSBTAG00000007307 | Fw: CAA TAC AGG TGT GGT AGG GAC | Simard et al., 2013 |
|  |  |  | Rv: TCA TGC CTT GCT AGG G |  |
| Tissue type plasminogen activator | PLAT | **ENSBTAG00000001244** | Fw: CCA CCC TGT TTT CAG CTA AAG | Simard et al., 2013 |
|  |  |  | Rv: TGC TGG GCT CTT GTG ACT |  |
| Urokinase plasminogen activator | PLAUR | ENSBTAG00000013125 | Fw: GCCCTACCAAATTGCTGTGT | García et al., 2018 |
|  |  |  | Rv: GCCGCAACGCTATCAATAAT |  |
| Trombospondin | THBS1 | ENSBTAG00000002006 | Fw: ATCATGGCTGACTCAGGAC | Farberov et al., 2014 |
|  |  |  | Rv: TAAGCCCATGGTTCCAGAA |  |
| Fibronectin | FN1 | ENSTAG00000000008300 | Fw : gcgattgtctgattctggctt | Guillomot et al., 2014 |
|  |  |  | Rv :tctccctgacgatcccactt |  |
| Metallothionein 1A | MT1A | ENSBTAG00000038067 | Fw: CACCTGCAAGGCCTGCAGA | Fujie et al., 2015 |
|  |  |  | Rv: CGAGGCCCCTTTGCAGACA  5`-CGAGGCCCCCTTTGCAGACA-3` |  |
| A disintegrin and metalloproteinase with thrombospondin motif | ADAMTS4 | ENSBTAG00000013210 | Fw: GCG-CCC-GCT-TCA-TCA-CTG | Behrendt et al., 2016 |
|  |  |  | Rv: TTG-CCG-GGG-AAG-GTC-ACG |  |
| A disintegrin and metalloproteinase with thrombospondin motif | ADAMTS1 | ENSBTAG00000013210 | Fw: CGGAAAAACCTTTAGAATGGAACA | Mishra et al., 2013 |
|  |  |  | Rv: AGGCCCGCTGCCAAA |  |
| Nuclear factor of kappa light polypeptide gene enhancer in B-cells 2 (p49/p100) | NFKB2 | ENSBTAG00000006017 | Fw: CCTGCTGAATGCTCTGTCTG | Magee et al 2012 |
|  |  |  | Rv: TCCTCCTTCACCTCTGTGCT |  |
| Glyderaldehyde 3-phosphate dehydrogenase | GAPDH | ENSBTAG00000014731 | Fw: ATCTCGCTCCTGGAAGATG  Rv: TCGGAGTGAACGGATTCG | Puech et al., 2015 |
| B-Actin | ACTB | ESBTAG00000026199 | Fw: ACACCGCAACCAGTTCGCCAT  Rv: GTCAGGATGCCTCTCTTGCT | Horcajo et al., 2017 |

**REFERENCES**

Arranz-Solís, D., Benavides, J., Regidor-Cerrillo, J., Horcajo, P., Castaño, P., del Carmen Ferreras, M., et al. (2016). Systemic and local immune responses in sheep after *Neospora caninumexperimental* infection at early, mid and late gestation. *Vet. Res*. 47:2. doi: 10.1186/s13567-015- 0290-0

Behrendt, P., Preusse-Prange, A., Klüter, T., Haake, M., Rolauffs, B., Grodzinsky, A. J., et al. (2016). IL-10 reduces apoptosis and extracellular matrix degradation after injurious compression of mature articular cartilage. *Osteoarth. Cartilage* 24, 1981–1988. doi: 10.1016/j.joca.2016.06.016

Farberov, S., and Meidan, R. (2014). Functions and transcriptional regulation of thrombospondins and their interrelationship with fibroblast growth factor-2 in bovine luteal cells. *Biol. Reprod*. 91:58. doi: 10.1095/ biolreprod.114.121020

Fujie, T., Murakami, M., Yoshida, E., Yasuike, S., Kimura, T., Fujiwara, Y., et al. (2016). Transcriptional induction of metallothionein by tris(pentafluorophenyl)stibane in cultured bovine aortic endothelial cells. *Int. J. Mol. Sci*.17:E1381. doi: 10.3390/ijms17091381

García, D. C., Russo-Maenza, A., Miceli, D. C., Valdecantos, P. A., and Roldán- Olarte,M. (2018). The urokinase plasminogen activator systemcomponents are regulated by vascular endothelial growth factor D in bovine oviduct*. Zygote*. 26, 242–249. doi: 10.1017/S0967199418000151

Gärtner, M. A., Peter, S., Jung, M., Drillich, M., Einspanier, R., and Gabler, C. (2016). Increased mRNA expression of selected pro-inflammatory factors in inflamed bovine endometrium in vivo as well as in endometrial epithelial cells exposed to Bacillus pumilus *in vitro. Reprod. Fertil. Dev*. 28, 982–994. doi: 10.1071/RD14219

Guillomot, M., Campion, E., Prézelin, A., Sandra, O., Hue, I., Le Bourhis, D., et al. (2014). Spatial and temporal changes of decorin, type I collagen and fibronectin expression in normal and clone bovine placenta. *Placenta* 35, 737–747. doi: 10.1016/j.placenta.2014.06.366

Jiménez-Pelayo, L., García-Sánchez, M., Regidor-Cerrillo, J., Horcajo, P., Collantes-Fernández, E., Gómez-Bautista, M., et al. (2019). Immune response profile of caruncular and trophoblast cell lines infected by high- (Nc-Spain7) and low-virulence (Nc-Spain1H) isolates of Neospora caninum. *Parasit. Vectors* 12:218. doi: 10.1186/s13071-019-3466-z

Ito, T., and Kodama, M. (1996). Demonstration by reverse transcriptionpolymerase chain reaction of multiple cytokine mRNA expression in bovine alveolar macrophages and peripheral blood mononuclear cells. *Res. Vet. Sci*. 60, 94–96.

Magee, D. A., Taraktsoglou, M., Killick, K. E., Nalpas, N. C., Browne, J. A., Park, S. D., et al. (2012). Global gene expression and systems biology analysis of bovine monocyte-derived macrophages in response to in vitro challenge with Mycobacterium bovis. *PLoS ONE* 7:e32034. doi: 10.1371/journal.pone.0032034

Mishra, B., Koshi, K., Kizaki, K., Ushizawa, K., Takahashi, T., Hosoe, M., et al. (2013). Expression of ADAMTS1 mRNA in bovine endometrium and placenta during gestation. *Domest. Anim. Endocrinol*. 45, 43–48. doi: 10.1016/j.domaniend.2013.04.002

Negrón-Pérez, V. M., Vargas-Franco, D., and Hansen, P. J. (2017). Role of chemokine (C-C motif) ligand 24 in spatial arrangement of the inner cell mass of the bovine embryo. *Biol. Reprod*. 96, 948–959. doi: 10.1093/biolre/iox037

Sakumoto, R., Hayashi, K.G., Fujii, S., Kanahara, H., Hosoe, M., Furusawa, T., et al. (2017). Possible roles of CC- and CXC-chemokines in regulating bovine endometrial function during early pregnancy. *Int. J Mol. Sci*. 18:E742. doi: 10.3390/ijms18040742

Simard, B., Ratel, D., Dupré, I., Pautre, V., and Berger, F. (2013). Shark cartilage extract induces cytokines expression and release in endothelial cells and induces E-selectin, plasminogen and t-PA genes expression through an antioxidant- sensitive mechanism. *Cytokine* 61, 104–111. doi: 10.1016/j.cyto.2012.08.035\

Garlanda, C., Dinarello, C., and Mantovani, A. (2013). The interleukin-1 family: back to the future. *Immunity* 39, 1003–1018. doi: 10.1016/j.immuni.2013.11.010
