# Supplementary material for: RNA-Seq Analyses Reveal That Endothelial Activation and Fibrosis Are Induced Early and Progressively by Besnoitia besnoiti Host Cell Invasion and Proliferation
Source: Front Cell Infect Microbiol. 2020 May 15;10:218. doi: 10.3389/fcimb.2020.00218 (PMC7242738; doi:10.3389/fcimb.2020.00218)
Supplement: Supplementary Table 2 — Primers used for RT-PCR validation of Besnoitia besnoiti genes. [file Table_2.DOC]

| **Gene name** | **Gene Symbol** | **Identifier** | **Primer sequence** | **Reference** |
| --- | --- | --- | --- | --- |
| MIC2 | MIC2 | maker-104-augustus-gene-0.27-mRNA-1 | Fw: TTACATGACGGGTTCGGC | This study |
|  |  |  | Rv: GCCTCCCTTTCTTCTTGCTC |  |
| MIC11 | MIC11 | maker-86-augustus-gene-0.75-mRNA-1 | Fw: TCGGAGAGAGTCTTTTCTCTGG | This study |
|  |  |  | Rv: TTTGAGAGCGTTCGTGAAGAC |  |
| GAP80 | GAP80 | augustus_masked-20-processed-gene-2.19-mRNA-1 | Fw: CCCTTCGCTATGTGCTTTTC | This study |
|  |  |  | Rv: CCCTCTTTCTCCGGAATCTC |  |
| ROP5B | ROP5B | augustus_masked-1-processed-gene-0.17-mRNA-1 | Fw: GGACGTAGAAACAGGCGAAG | This study |
|  |  |  | Rv: GAGCTTCTTGAGGGGTGTTG |  |
| ROP17 | ROP17 | augustus_masked-24-processed-gene-1.27-mRNA-1 | Fw: GGAGGCAGCAGGAACATTAG | This study |
|  |  |  | Rv: TCATTCGTTTCGAGTGCAAG |  |
| ROP40 | ROP40 | augustus_masked-15-processed-gene-3.1-mRNA-1 | Fw: CGGCTGTCTCCTTCTTTGTC | This study |
|  |  |  | Rv: GCCCTGTCTATTGCTTCGAC |  |
| SRS22A | SRS22A | maker-68-augustus-gene-0.65-mRNA-1 | Fw: TTACCTGGTTCTTCGGCG | This study |
|  |  |  | Rv: TTGACTGCTGGAGGGAGAG |  |
| GRA7 | GRA7 | augustus_masked-38-processed-gene-1.18-mRNA-1 | Fw: CGTTGTGCTTGCTAGACCTG | This study |
|  |  |  | Rv: TCCTCGTCCACATCCTCTTC |  |
| GRA10 | GRA10 | maker-60-augustus-gene-0.22-mRNA-1 | Fw: TCTGCGAAAAAGGAGACCC | This study |
|  |  |  | Rv: GCACTTCACCATTCTGGTCC |  |
| PEP | PEP | maker-42-augustus-gene-1.23-mRNA-1 | Fw: CGCTCTTCACTGTCGCATC | This study |
|  |  |  | Rv: CGGAAAGAAACGAGAAGACG |  |
| SRS | SRS | augustus_masked-99-processed-gene-0.18-mRNA-1 | Fw: TGAGAAAGCAACGTCCAGTG | This study |
|  |  |  | Rv: CCAGAAGCCGAGAAGACAAG |  |
| ACT | ACT | maker-12-augustus-gene-3.48-mRNA-1 | Fw: GAACCCCGGTATCATGGTGG | This study |
|  |  |  | Rv: TACCTCTCTTGCTCTGGGCT |  |
| GAPDH | GAPDH | maker-41-augustus-gene-5.43-mRNA-1 | Fw: GGCGTGGAAGTTGTTGCTATC | This study |
|  |  |  | Rv: CCGTGAACGGAGTCGTACTTC |  |
